# Learning to reverse thermal diffusion

**DOI:** 10.1093/nsr/nwag287

**Published:** 2026-05-15

**Authors:** Hanqi Chen, Qiang-Kai-Lai Huang, Yanxiang Wang, Yifan Shou, Pei-Chao Cao, Wenduo Yu, Dong Wang, Zhun Wei, Hongsheng Chen, Jiping Huang, Ying Li

**Affiliations:** International Joint Innovation Center, The Electromagnetics Academy, Zhejiang University, Haining 314400, China; State Key Laboratory of Extreme Photonics and Instrumentation, Zhejiang Key Laboratory of Intelligent Electromagnetic Control and Advanced Electronic Integration, Zhejiang University, Hangzhou 310027, China; Jinhua Institute of Zhejiang University, Zhejiang University, Jinhua 321099, China; International Joint Innovation Center, The Electromagnetics Academy, Zhejiang University, Haining 314400, China; State Key Laboratory of Extreme Photonics and Instrumentation, Zhejiang Key Laboratory of Intelligent Electromagnetic Control and Advanced Electronic Integration, Zhejiang University, Hangzhou 310027, China; Jinhua Institute of Zhejiang University, Zhejiang University, Jinhua 321099, China; International Joint Innovation Center, The Electromagnetics Academy, Zhejiang University, Haining 314400, China; State Key Laboratory of Extreme Photonics and Instrumentation, Zhejiang Key Laboratory of Intelligent Electromagnetic Control and Advanced Electronic Integration, Zhejiang University, Hangzhou 310027, China; Jinhua Institute of Zhejiang University, Zhejiang University, Jinhua 321099, China; International Joint Innovation Center, The Electromagnetics Academy, Zhejiang University, Haining 314400, China; State Key Laboratory of Extreme Photonics and Instrumentation, Zhejiang Key Laboratory of Intelligent Electromagnetic Control and Advanced Electronic Integration, Zhejiang University, Hangzhou 310027, China; Jinhua Institute of Zhejiang University, Zhejiang University, Jinhua 321099, China; International Joint Innovation Center, The Electromagnetics Academy, Zhejiang University, Haining 314400, China; State Key Laboratory of Extreme Photonics and Instrumentation, Zhejiang Key Laboratory of Intelligent Electromagnetic Control and Advanced Electronic Integration, Zhejiang University, Hangzhou 310027, China; Jinhua Institute of Zhejiang University, Zhejiang University, Jinhua 321099, China; Hangzhou International Innovation Institute, Beihang University, Hangzhou 311115, China; International Joint Innovation Center, The Electromagnetics Academy, Zhejiang University, Haining 314400, China; State Key Laboratory of Extreme Photonics and Instrumentation, Zhejiang Key Laboratory of Intelligent Electromagnetic Control and Advanced Electronic Integration, Zhejiang University, Hangzhou 310027, China; Jinhua Institute of Zhejiang University, Zhejiang University, Jinhua 321099, China; International Joint Innovation Center, The Electromagnetics Academy, Zhejiang University, Haining 314400, China; State Key Laboratory of Extreme Photonics and Instrumentation, Zhejiang Key Laboratory of Intelligent Electromagnetic Control and Advanced Electronic Integration, Zhejiang University, Hangzhou 310027, China; Jinhua Institute of Zhejiang University, Zhejiang University, Jinhua 321099, China; International Joint Innovation Center, The Electromagnetics Academy, Zhejiang University, Haining 314400, China; State Key Laboratory of Extreme Photonics and Instrumentation, Zhejiang Key Laboratory of Intelligent Electromagnetic Control and Advanced Electronic Integration, Zhejiang University, Hangzhou 310027, China; International Joint Innovation Center, The Electromagnetics Academy, Zhejiang University, Haining 314400, China; State Key Laboratory of Extreme Photonics and Instrumentation, Zhejiang Key Laboratory of Intelligent Electromagnetic Control and Advanced Electronic Integration, Zhejiang University, Hangzhou 310027, China; Jinhua Institute of Zhejiang University, Zhejiang University, Jinhua 321099, China; Department of Physics, State Key Laboratory of Surface Physics, Fudan University, Shanghai 200438, China; Key Laboratory of Micro and Nano Photonic Structures (Ministry of Education), Fudan University, Shanghai 200438, China; School of Physics, Faculty of Basic Sciences, University of Shanghai for Science and Technology, Shanghai 200093, China; International Joint Innovation Center, The Electromagnetics Academy, Zhejiang University, Haining 314400, China; State Key Laboratory of Extreme Photonics and Instrumentation, Zhejiang Key Laboratory of Intelligent Electromagnetic Control and Advanced Electronic Integration, Zhejiang University, Hangzhou 310027, China; Jinhua Institute of Zhejiang University, Zhejiang University, Jinhua 321099, China

**Keywords:** heat transfer, thermal field, inversed thermal diffusion, operator learning

## Abstract

Thermal diffusion, which governs heat transfer across a wide range of systems—from electronics to industrial processes—is inherently irreversible under the second law of thermodynamics, thus obscuring time-dependent information. To overcome this ill-posedness, this study introduces a physics-informed framework that centers on a novel time-reversal operator learning approach. A finite-difference-based network is first employed to robustly derive heterogeneous material properties. Crucially, the core innovation lies in the operator for thermal retrodiction. Distinct from traditional point-wise solvers, this functional model learns the mapping between function spaces, enabling the direct projection of the final-state thermal field back to its initial state. By synergizing analytical eigenbasis decomposition with frequency-domain operator learning, the time-reversal operator effectively reconstructs the backward propagation of temperature fields. Validated on 3D-printed structures and chips, this operator-driven method achieves retrodiction errors $ < $0.1%, establishing a high-fidelity paradigm for spatiotemporal analysis. This breakthrough has broad implications for non-destructive testing in energy systems, with potential applications extending to a wide class of phenomena such as mass, charge, and light diffusion.

## Teaser

This work reports a deep learning framework for inverse thermal diffusion, accurately deducing initial thermal states from final-state observations to advance non-destructive testing and thermal management.

## INTRODUCTION

Understanding and controlling the temporal evolution of physical processes is central to science and engineering. While predicting future states (forward problems) has seen immense success, inferring past states from current observations (inverse problems) presents significant challenges due to their inherent ill-posedness and sensitivity to noise. Thermal diffusion, a fundamental physical process pervasive in both natural and engineered systems, exemplifies this challenge. It is critical for applications ranging from electronic device thermal management to industrial non-destructive testing. In semiconductor technologies, for instance, the miniaturization and increasing power densities of integrated circuits require advanced thermal regulation strategies to ensure device reliability and performance. However, modeling heat transfer in complex structures, particularly those involving heterogeneous materials and complex boundary conditions, poses significant challenges for conventional methods.

Recent years have seen advancements in thermal field management strategies, notably through innovations in material design. The emergence of thermal metamaterials [1,2] for example, has opened new avenues for thermal regulation. Originally based on coordinate transformation theory, these materials have enabled the design of functional thermal devices [3]. Other considerations in thermal regulation, such as non-reciprocity [4], topological heat transport [5–7] and non-Hermitian thermal metamaterials [8,9] have been proposed to date. Wang *et al.* observed the macroscale anomalous thermal diffusion for the first time in the active thermal metamaterials with programmed heat and cold sources, which offers a refreshing and promising new direction for advanced thermal management [10]. Furthermore, there is a growing interdisciplinary field bridging thermal fields with other subjects, such as thermo-electrics [11], thermo-mechanics [12], thermo-photonics [13] and machine learning-aided metamaterials [14–17]. While significant progress has been made in thermal metamaterials, addressing complex practical challenges remains difficult. Existing research primarily focuses on spatial parameters, overlooking the valuable information contained within the temporal dimension of thermal fields, which conventional frameworks find particularly challenging to analyze.

Fortunately, the rapid advancement of deep learning techniques provides powerful tools for solving complex thermal diffusion problems in both spatial and temporal dimensions. Specifically, methods like Diffusion Models [18–20] and Operator Learning [21,22] have opened new avenues for addressing retrodiction problems. However, the physical nature of thermal diffusion itself makes the inverse process extremely challenging. Thermal diffusion is an inherently dissipative process governed by parabolic partial differential equations (PDEs), in which temperature gradients spontaneously smooth out over time. As a result, detailed information about the initial thermal distribution is irreversibly lost as entropy increases. This information loss leads to a severe ill-posedness in the inverse (time-reversal) problem: infinitesimal errors in the final temperature field can cause enormous uncertainties in the reconstructed initial state. Despite this intrinsic difficulty, solving the ‘thermal field retrodiction’ problem is of great scientific and practical significance. Accurate reconstruction of past thermal states can reveal hidden physical processes, enable nondestructive diagnostics in material testing, and improve thermal management in electronics and energy systems. For instance, diffusion models have been successfully employed for the inverse design of periodic lattice structures in thermal metamaterials [23], effectively mitigating the ill-posed nature of the retrodiction. Simultaneously, embedding physical governing equations into neural networks [23–25] has significantly reduced the dependence on large-scale datasets. Nevertheless, existing methods still face several limitations: data-driven models often lack interpretability without physical constraints, and most studies focus on homogeneous materials or simplified scenarios, making direct generalization to practical applications in complex structures challenging [26].

**Figure 1.**
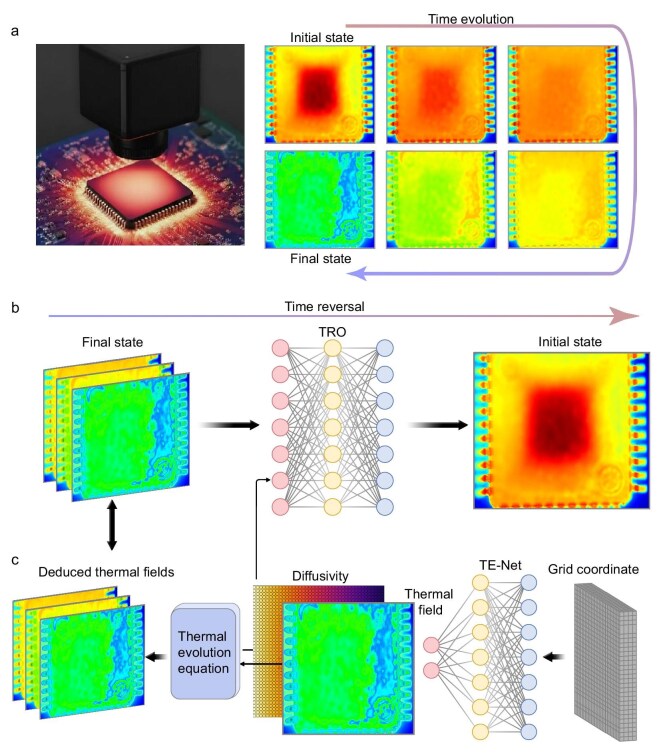
Schematic of the diffusion and retrodictive diffusion processes, and the overall deep learning structure. (a) The image shows the thermal evolution of a chip under load captured by a thermal imaging camera. The right image depicts the thermal map evolution of the chip during thermal diffusion. (b) The thermal evolution data of the final state is fed into the TRO network to retrodict the initial thermal field. (c) Using mesh information, the thermal diffusivity is estimated based on the thermal field evolution data, as shown in the TE-Net diagram. The inferred thermal field is then compared with the actual field, and the obtained thermal diffusivity is subsequently fed into the TRO to retrodict the initial temperature field.

Here, we introduce a novel thermal field retrodiction framework that, for the first time, synergistically combines a physics-constrained parameter inference network with a prior-knowledge-guided time-reversal operator (TRO) learning approach. This framework is specifically designed to overcome the shortcomings of conventional methods when dealing with heterogeneous materials and retrodicting complex thermal fields (depicted in Fig. 1a). Our methodology consists of two main components. First, we introduce the Thermal Field Evolution Network (TE-Net), shown in Fig. 1c, which accurately estimates the material parameters, particularly diffusivity, from cooling data. Second, we leverage the strengths of both diffusion models and operator learning to develop a TRO based on Discrete Cosine Transform (DCT) correction with its workflow illustrated in Fig. 1b. Critically, the diffusivity estimated by TE-Net serves as essential prior knowledge for the TRO. Both models fully utilize the inherent temporal information within the diffusion process, enabling more effective parameter quantification in complex dissipative processes and highly accurate retrodiction of the initial thermal field. Through comprehensive simulations and experiments, our method achieves a thermal diffusivity estimation error of $ < $10$ \% $ and a numerical error of $ < $0.1$\%$ for thermal field retrodiction, demonstrating its high fidelity in reconstructing complex initial thermal states.

## RESULTS

### Derivation of thermal parameters

For a certain non-uniform material, heat flow consists of two parts: conduction and convection. The conduction part comes from the surrounding area of the test region, while the convection part arises from the advection between the material and the air. Thus, the governing equation is


**Figure 2.**
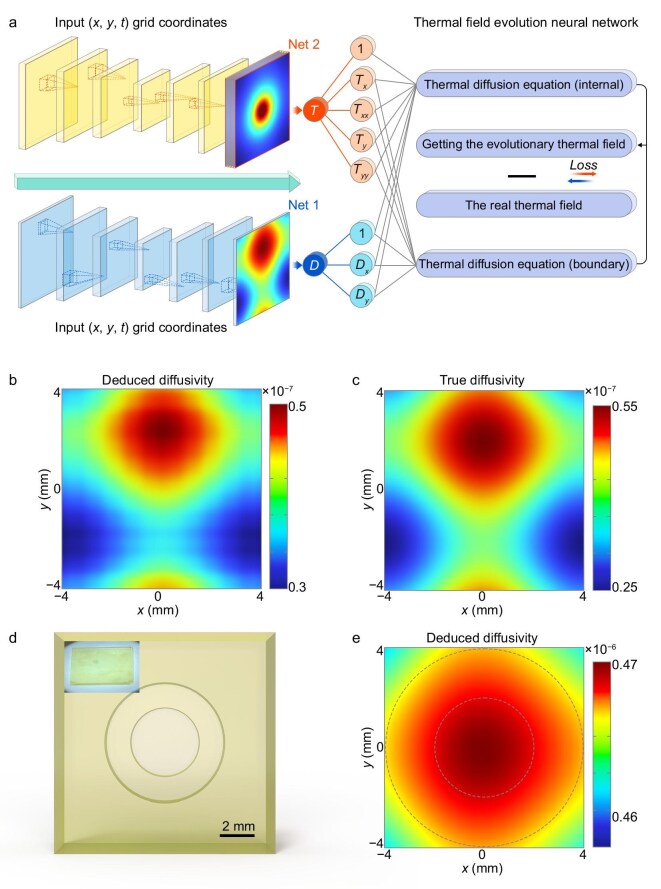
Thermal parameters inference network and the simulation/experiment results. (a) Net2 (upper panel) is used to derive the time derivative for the diffusion equation, while Net1 (bottom panel) is responsible for deriving the diffusivity and spatial partial derivatives for the diffusion equation. The combination of these two networks, along with the diffusion equation, allows for the derivation of the temperature evolution, which is then compared to the actual thermal field evolution. (b) Predicted thermal diffusivity distribution based on the simulation dataset. (c) Actual thermal diffusivity distribution in the simulation. (d) A sample printed using 3D printing technology, featuring an externally enclosed structure with two concentric holes in the center. The top right inset shows a physical image of the sample. (e) Inferred thermal diffusivity distribution of sample shown in (d).

(1) \begin{eqnarray*}
\rho c_p\frac{\partial T}{\partial t}=\nabla \cdot \left(\kappa \nabla T\right)+\frac{h}{\Delta z}\left(T_{\rm e}-T\right),
\end{eqnarray*}

where $\rho$, $c_p$, $T$, $t$, $\kappa$, $h$, $T_{\rm e}$ and $\Delta z$ represent density, specific heat capacity at constant pressure, temperature, time, thermal conductivity, Newton’s cooling coefficient, ambient temperature and characteristic length in the $z$-direction, respectively. The thermal diffusivity is defined as $D = \kappa \rho ^{-1}c_p^{-1}$. In our problem, we assume that the thermal diffusivity is independent of temperature and time. Therefore, we only need to estimate the geometric distribution of material parameters, which can be achieved using Net1 in TE-Net (Fig. 2a, bottom panel). However, according to Equation (1), if we consider the material parameters as the variables to be solved, both the time and geometric partial derivatives of temperature must be given. Net1 in Fig. 2a has geometric input $(x, y)$, so the geometric partial derivatives can be calculated. But we also need another network capable of computing time derivative. Based on this, we additionally build net2, which contains temporal dynamics (Fig. 2a, upper panel), providing a time derivative for net1 to estimate diffusivity (Note S1). Note that the diffusivity ($D = \kappa \rho ^{-1}c_p^{-1}$) is the main parameter we need to estimate. However, another unknown parameter, $h\rho ^{-1}c_p^{-1}$ exists. Through simulation and experiment, we found that this parameter has a weak effect on the inference of time retrodiction of thermal field. Therefore, $h\rho ^{-1}c_p^{-1}$ is treated as a single parameter to be iterated slowly in our model, which is initialized with a generalized empirical value based on physical prior knowledge, and subsequently updated through backpropagation alongside the other network parameters. Slow integration describes the observed convergence behavior where it functions as a ‘slow variable’ relative to other parameters rather than a distinct algorithmic constraint.

Regarding the specific structure of the networks, previous studies on physics-informed neural network (PINN) have predominantly employed fully connected neural networks (FCNNs) for modeling such problems [27–29]. However, when using FCNNs, the spatial coordinates of the computational grid are flattened into vectors, leading to a loss of spatial correlations, which weakens the ability to solve anisotropic problems. In this study, we adopt a convolutional neural network (CNN), which utilizes convolution operations to extract spatial features from the input data, enabling TE-Net to effectively learn the spatial interdependencies of the grid points. At this stage, TE-Net successfully derived a set of high-precision diffusivity distributions. This accurate diffusivity information, precisely obtained from TE-Net, serves as crucial physical prior knowledge for the subsequent TRO model, laying the foundation for a unified and self-consistent thermal field retrodiction framework that significantly enhances overall retrodiction accuracy and stability.

However, we cannot assess the accuracy of our estimations, as we have not measured the actual material parameters for our samples, and testing them is difficult. On the other hand, the temperature field distribution is more intuitive for testing. We can make full use of time-dependent temperature evolution data, which is easier to obtain, to evaluate the accuracy of material parameter estimates. Given that a physical model can make the entire evolution process more interpretable and accurate, the discrete heat transfer equations are introduced to generate temperature evolution data based on the material parameters deduced from net1. According to Equation (1), the thermal transfer equations for both the bulk area and boundaries (using the left boundary as an example) can be written as


(2) \begin{eqnarray*}
T_{m,n}^{(t+1)} &=& \frac{\Delta t}{\rho c_p}\biggl [ \kappa \frac{T_{m+1,n}^{(t)}+T_{m-1,n}^{(t)}-2T_{m,n}^{(t)}}{(\Delta x)^2}\\
&&+\, \kappa \frac{T_{m,n+1}^{(t)}+T_{m,n-1}^{(t)}-2T_{m,n}^{(t)}}{(\Delta y)^2}\\
&&+\, \frac{h(T_e-T_{m,n}^{(t)})}{\Delta z}\biggr ] + T_{m,n}^{(t)},
\end{eqnarray*}

(3) \begin{eqnarray*}
T_{m,n}^{(t+1)} &=& \frac{\Delta t}{\rho c_p}\biggl [ \frac{h(T_{\rm e}-T_{m,n}^{(t)})}{\Delta x} + \kappa \frac{T_{m+1,n}^{(t)}-T_{m,n}^{(t)}}{(\Delta x)^2} \\
&& +\, \kappa \frac{T_{m,n+1}^{(t)}+T_{m,n-1}^{(t)}-2T_{m,n}^{(t)}}{(\Delta y)^2}\\
&&+\, \frac{h(T_e-T_{m,n}^{(t)})}{\Delta z}\biggr ] + T_{m,n}^{(t)}.
\end{eqnarray*}

For the expression $T_{m,n}^{(t)}$, the superscript ($t$) represents the time $t$  $\times$  $\Delta t$, the subscript $m$ corresponds to the $m$th pixel in the $x$-direction, and $n$ corresponds to the $n$th pixel in the $y$-direction. $T_{\rm e}$ denotes the ambient temperature, which can be assumed constant in this system. $h$ is Newton cooling coefficient. Additionally, $\Delta z$ corresponds to the thickness in the $z$-direction, and $\Delta x$, $\Delta y$ denote the grid sizes in $x$- and $y$-directions, respectively. Using this approach, other boundary evolution equations can be derived. Through this formulation, the evolution of the object during the cooling process can be fully characterized, guiding the model to infer the correct distribution of the heat diffusion coefficient. Crucially, integrating these explicit boundary constraints is essential to prevent cumulative errors in the thermal diffusivity derivation from propagating throughout the time-dependent thermal field inversion. The overall process is illustrated in Fig. 2a. For physical derivation process, see Notes S2 and S3.

Theoretically, based on the equations above, knowing the temperature field at $t = 0$ and a set of material parameters is sufficient to predict the temperature field at any subsequent time. To evaluate the model’s performance, we compare the inferred temperature field with the ground truth and define the loss function as follows:


(4) \begin{eqnarray*}
{Loss}=\frac{1}{N^2t_{\mathrm{total}}}\sum _{t=0}^{t_{\mathrm{total}}}\sum _{m=1}^{N}\sum _{n=1}^{N}\left(T_{m,n}^{(t)}-\left.T_{m,n}^{\left(t\right)}\right|_\mathrm{real}\right)^2,\!\!\!\\
\end{eqnarray*}

where $t_\mathrm{total}$ is the testing time for the whole process, and $N$ is the total number of grids in both the $x$ and $y$ directions. Based on this loss function, reverse propagation iteration is operated to obtain the optimal material coefficient. The theoretical derivations above assume a quasi-two-dimensional model. However, in practice, the targets detected are three-dimensional structures. Since infrared cameras can only capture data on two-dimensional surfaces, obtaining three-dimensional data with sensors is costly. In this work, the heat conduction in the $z$-direction is reflected on the two-dimensional plane through the modification of material parameters. Since the prior knowledge of material parameters is not provided, it is applicable to use the two-dimensional thermal field to deduce material parameters of three-dimensional structure [30]. When the specimen possesses a moderate thickness (thermally thin), the effective surface thermal conductivity can be analytically derived.

### Simulation and experiment for estimating material parameters

We use COMSOL Multiphysics simulation software to verify the theoretical feasibility of the model. Since laser heating is employed in the real experiment, a point heat source is used in the simulation as its counterpart. To obtain representative results inferred from the model, we define the thermal conductivity of the model as varying cosine in the $x$-direction and sinusoidal in the $y$-direction. The specific expression is as follows:


(5) \begin{eqnarray*}
\kappa =\kappa _0 +A_1\cos \!{\left(\frac{2\pi x}{L}\right)}+A_2\sin {\left(\frac{2\pi y}{L}\right)}.
\end{eqnarray*}

The thermophysical parameters of sample are $A_1 = A_2 = 2$, $L=8\,\mathrm{mm}$, $\kappa _0 = 10\,{\mathrm{W \, m^{-1} \, K^{-1}}}$, $\rho = 6000\,\,\,\mathrm{kg \, m^{-3}}$, and specific heat $c_p = 420\,\,\,\mathrm{J \, kg^{-1} \, K^{-1}}$. We performed a simulation for $10\,{\mathrm{s}}$, with the heating time of the heat source set to $5\,\mathrm{s}$. The time step $\Delta t$ is $0.1\,{\mathrm{s}}$. The total dimensions of the object are $8\,\mathrm{mm} \times 8\,\mathrm{mm} \times 1\,\mathrm{mm}$, and the dimensions of each discrete voxel are $\Delta x = \Delta y = 0.08\,\mathrm{mm}$.

During the simulation, we define a coordinate grid on the upper surface of the rectangular object and only use the surface temperature to train our model. Since the thermal camera captures only surface temperature, we use these simulated surface profiles as real values to train and test the model’s performance. The inferred thermal diffusivity distribution and the actual thermal diffusivity distribution are shown in Fig. 2b and c, respectively, matching closely to each other.

**Figure 3.**
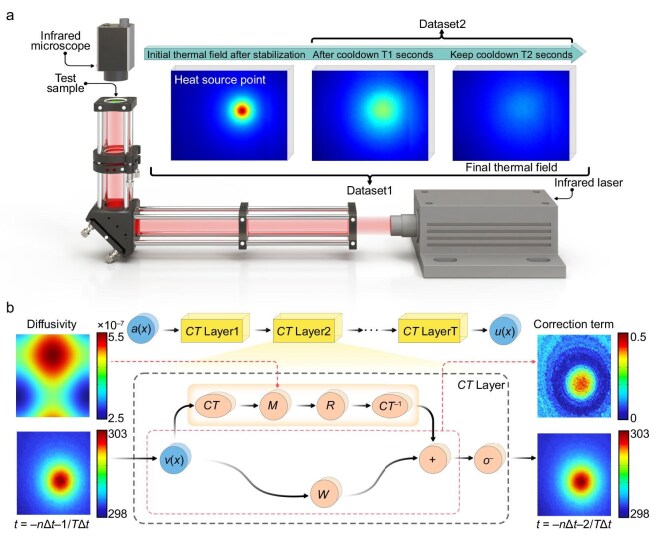
The experimental setup and model architecture. (a) An infrared laser is directed from below to heat the sample through a prism. Once thermal equilibrium is reached, an infrared microscope is used to record the temperature evolution on the up surface. The initial thermal field can be adjusted by changing the laser’s position. (b) The thermal diffusivity obtained from Fig. 2b is embedded into the TRO model. The bottom left and bottom right images show the thermal fields before and after passing through one module, respectively. The upper right image shows the correction term *W*.

For the experimental part, given the high degree of integration of chips, with most dimensions on the millimeter scale or even smaller, we observe the thermal field evolution of the object under test using an infrared microscope, with each pixel of the microscope measuring $20 \ \mu \mathrm{m} \times 20 \ \mu \mathrm{m}$. The object under observation is a perforated structure, sizing 8 mm $\times$ 8 mm, with concentric circles perforated at the center through 3D printing, as shown in Fig. 2d. The internal holes in the sample effectively create a heterogeneous surface diffusivity distribution. Dataset1 in Fig. 3 is transferred into the model, which helps to derive thermal diffusivity distribution (Fig. 2e). We clearly observe that the thermal diffusivity distribution consists of two concentric circles with different radii, and the values are similar to those of the thermal diffusivity of photosensitive resin. This demonstrates that our network can accurately infer the thermal diffusivity distribution of the observed object.

### Diffusion retrodiction

In this section, we propose a prior-guided TRO method for thermal field retrodiction. The diffusivity derived from TE-Net in the preceding steps serves as prior knowledge, which is then used as input for the TRO deep learning model. This model leverages the final-state temperature field and material parameters to retrodict the initial temperature distribution.

We draw inspiration from generative diffusion models in machine learning, which generate data by learning to reverse a stochastic noise process. Similarly, our TRO aims to learn to reverse a physical dissipation process. However, unlike the former, the TRO is an operator constrained by physical parameters (diffusivity).

We define an evolution equation to learn the retrodiction diffusion process of time-dependent thermal fields. By establishing such a framework, the initial thermal state can be inferred from the final temperature field. Starting with a parameter-free heat conduction PDE, we express the system as an evolution equation:


(6) \begin{eqnarray*}
u\left(x,y,t\right)=F\left(t\right)u\left(x,y,t_0\right),
\end{eqnarray*}

where $F(t)$ is the evolution operator. An operator maps functions from one space to another. In neural networks, convolutional layers, fully connected layers, and similar components act as operators. Unlike PINNs, which sample input-output pairs from a given function, our operator aims to learn mappings between function spaces (e.g., from final-state to initial-state thermal fields). The Graph Neural Operator [31] provides a powerful framework for solving PDEs by introducing the Green’s-function-like operator. However, real-world thermal evolution inevitably involves noise, which hinders the accuracy of inversion operator. To address this, we introduce a correction term *W*, inspired by the Fourier Neural Operator (FNO) [32], and incorporate it into the evolution equation. The update rule from $v_{k}$ to $v_{k+1}$ is defined as


(7) \begin{eqnarray*}
v_{k+1}(x) := \sigma \left[W v_k(x) + (\mathcal {K}(a;\phi ) v_k)(x)\right].
\end{eqnarray*}

Here, let $D\subset \mathbb {R}^d$ be a bounded, open set and $\mathcal {A}=\mathcal {A}\left(D;\mathbb {R}^{d_a}\right)$ and $\mathcal {U}=\mathcal {U}\left(D;\mathbb {R}^{d_v}\right)$ be separable Banach spaces of function taking values in $\mathbb {R}^{d_a}$ and $\mathbb {R}^{d_v}$ respectively. $a\in \mathcal {A}$ and $x$ is discretized at intervals of $\Delta x$. The operator $\mathcal {K}:\mathcal {A}\times \mathrm{\Theta }_\mathcal {K}\rightarrow \mathcal {L}\left(\mathcal {U}\left(D;\mathbb {R}^{d_v}\right),\mathcal {U}\left(D;\mathbb {R}^{d_v}\right)\right)$ maps to bounded linear operators on $\mathcal {U}\left(D;\mathbb {R}^{d_v}\right)$ and is parameterized by $\phi \in \mathrm{\Theta }_\mathcal {K}$. $\mathrm{\Theta }_\mathcal {K}$ is finite-dimensional parameter space. Additionally, $\mathrm{W }\!\!:\mathbb {R}^{d_v}\rightarrow \mathbb {R}^{d_v}$ represents a linear transformation, and $\sigma \!:\mathbb {R}\rightarrow \mathbb {R}$ is a non-linear activation function whose action is defined component-wise. We select $\mathcal {K}\left(a;\phi \right)$ to be a kernel integral transformation parameterized by a neural network. This serves as the counterpart to the evolution operator $F(t)$ in Equation (6).

Returning to Equation (6), the evolution operator $F(t)$ can be explicitly derived under Neumann boundary conditions using operator exponential functions [33]:


(8) \begin{eqnarray*}
u(x,y,t) &=& \sum _{n=0}^{\infty }\sum _{m=0}^{\infty } A_{nm} \exp \biggl [-\pi ^2 \\
&&\times \, \left(\frac{n^2}{W^2} + \frac{m^2}{H^2}\right)(t-t_0)\biggr ] \\
&&\times \, \cos \left(\frac{n\pi x}{W}\right) \cos \left(\frac{m\pi y}{H}\right),
\end{eqnarray*}

where $A_{nm}$ are coefficients of the initial state $u(x,y,t_0)$ projected onto eigenfunctions $\varphi _{n,m}(x,y)$, and $\lambda _{n,m} = \pi ^2(n^2/W^2+m^2/H^2)$ are the corresponding eigenvalues. Under this cosine basis, thermal field evolution can be described through eigenvalue dynamics. By mapping the kernel integral operator to the frequency domain, the physical interpretability of the model is enhanced. To incorporate material parameters, specifically thermal diffusivity *D* derived from TE-Net in the preceding steps, we update Equation (7) as


(9) \begin{eqnarray*}
v_{k+1} && := \sigma \Bigl \lbrace Wv_k(x) + CT^{-1}\Bigl [ \\
&& e^{\lambda _{n,m}(t-t_0)}CT(D)\cdot CT(v_k) \Bigr ](x) \Bigr \rbrace .
\end{eqnarray*}

where $CT$ and $CT^{-1}$ denote the cosine transform and its inverse, respectively. Since thermal energy predominantly resides in low-frequency components, high-frequency noise is filtered out to retain essential information. This formulation embeds physical principles into the model architecture (Fig. 3b), improving interpretability and computational efficiency. For detailed evolution derivation, see Note S4.

### Numerical and experimental validation

**Figure 4.**
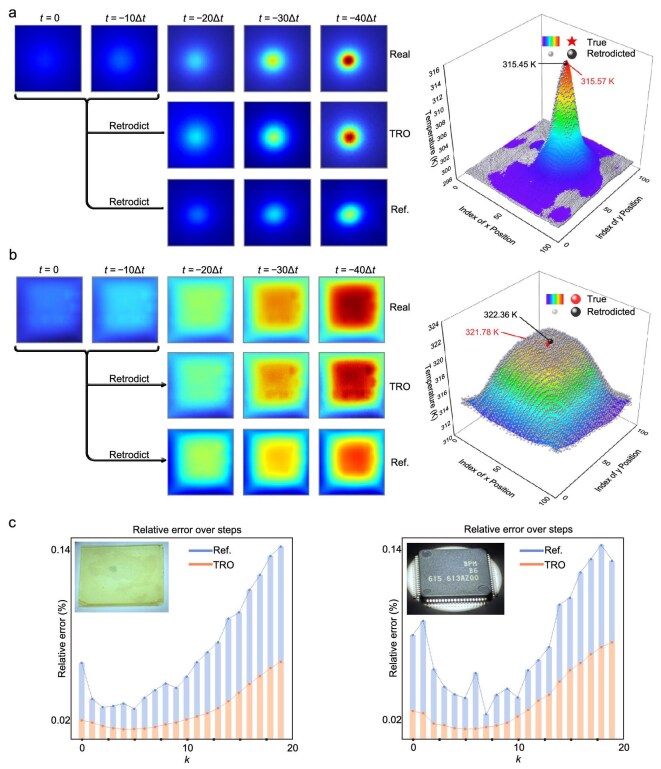
Experimental results with different samples. (a) The thermal field retrodiction evolution of a 3D-printed concentric perforated structure. The images compare the actual thermal field with the retrodiction thermal field predicted by our model and Ref. model at corresponding time steps. (b) The thermal field retrodiction of a real chip. The images compare the actual thermal field with the thermal fields predicted by our model and Ref. model at corresponding time steps. (c) Relative error with increasing time step for different detection targets in Fig. 4a and b, respectively.

We first validate our TRO retrodiction model using the diffusivity dataset in Fig. 2b, which serves as the basis of numerical verification. We select one layer and compare the thermal field at the input with the next step after a single iteration. The results are shown in Fig. 4a, with the correction term $W$ plotted in the upper right corner. It can be observed that merely using eigenvalues to predict the evolved thermal field introduces errors compared to the actual thermal field. To address this, we introduce a learnable residual correction term $W$, primarily designed to compensate for discrepancies arising during the process of deriving three-dimensional material parameters from a two-dimensional thermal field using TE-Net, as well as for simultaneous environmental energy exchange and contact between the sample and substrate. As shown in Fig. 3b, we visualize the learned $W$ term, whose spatial distribution exhibits a notable correlation with changes in the model’s boundary conditions and regions of local material heterogeneity. This indicates that *W* is not merely a ‘fudge factor’ but a physically meaningful compensation term capable of capturing and correcting complex multiphysical coupling effects. After applying this correction, the experimental error is reduced by $\sim$10$\%$, demonstrating its significance.

Beyond simulation, we further retrodict the actual thermal field of different detection objects. The dataset is the dataset2 in Fig. 3a. First, we examine a perforated structure made of photosensitive resin via 3D printing (Fig. 4a). Its low thermal conductivity inherently limits lateral heat diffusion, thereby ensuring confinement of the thermal energy to a well-defined point heat source. The retrodicted results align closely with the actual thermal distribution, accurately capturing the point heat source. Furthermore, we assess the model’s generalizability in a practical chip as the testing object. Figure 4b reveals that, due to its inherent structure, the chip’s actual heat source deviates from an idealized point-like one. Additionally, the retrodicted results in Fig. 4b highlight localized heat sources with high accuracy, demonstrating the effectiveness for subsequent heat source detection. The experimental setup and dataset can be found in the ‘Materials and Methods’ section. Training details can be found in Note S5.

In addition, in order to illustrate the superiority in retrodicting thermal field, we also perform control experiments compared with a reference model (Ref. model) that is not based on prior knowledge. Figure 4b and c simultaneously exhibit the retrodicted results between TRO and Ref. model, according to real thermal field. The reference model is the standard FNO architecture, which learns mappings between function spaces in the frequency domain but lacks physical constraints. We calculate the error between the retrodicted results and the real thermal field, and plot two error curves over time steps (Fig. 4c), where the error value is partitioned from the main heat source evolution region based on the real heat field map (Note S6). Clearly, although relative error increases physically, the value of TRO is obviously smaller than that of Ref. model at each step. Detailed architecture of the reference model is provided in Note S7.

## DISCUSSION

This study proposes a novel physics-informed operator learning, time-dependent thermal field retrodiction method that integrates deep learning with physics-based modeling to address the challenges of inferring thermal properties in complex heterogeneous materials and retrodicting the temperature field distribution at the initial state. The proposed approach demonstrates three core contributions: (1) a physics-informed architecture combining CNNs with operator model in cosine basis, enabling high-precision retrodiction of initial heat source distributions and thermal parameters with spatial-temporal fidelity; (2) systematic validation through numerical simulations and infrared thermal imaging experiments, showing $< 10\%$ error in thermal diffusivity estimation and $< 0.1\%$ numerical error in thermal field retrodiction; and (3) a primary demonstration of practical applicability in critical engineering scenarios like chip heating detection. The proposed retrodiction method also offers potential for developing advanced countermeasures against thermal side-channel attacks.

The accuracy of the proposed method is validated through simulations of local thermal field evolution driven by various heat sources, supported by experimental data obtained from infrared thermal imaging. Furthermore, the integration of operator learning with physics-based modeling. enhances the robustness of the inference results, effectively reduces noise interference, and improves the accuracy of time-varying thermal field retrodiction. It better preserves spatial information and enables precise learning of spatiotemporal temperature field dynamics, compared with CNNs.

Extensive validation through both simulation experiments and practical applications demonstrates that the proposed time-dependent retrodiction model, based on final-state temperature field data, accurately reconstructs thermal field distributions. The method shows significant potential for applications in materials science, engineering testing, and industrial non-destructive testing. This innovative approach bridges the gap between deep learning and physical modeling, offering an efficient and accurate tool for thermal field analysis and defect detection in practical engineering scenarios. By addressing limitations in traditional numerical simulations, this work paves the way for advanced thermal diagnostics and optimization in complex material systems.

## MATERIALS AND METHODS

To simulate the heat generation process and accurately replicate the occurrence of an abnormal heat source in a real-world scenario, as illustrated in Fig. 3, we employ a continuously emitting laser to mimic a localized point heat source within an object. The laser operates in the infrared spectrum and is directed through a prism to alter its optical path, ensuring that it strikes the test object perpendicularly from the bottom surface. A lens is then used to focus the laser, creating a concentrated point heat source. Experimental measurements confirm that the actual laser spot diameter is $< $1 mm, with propagation losses in the transmission path kept $< $5$\%$, effectively simulating the localized heating effect caused by defects in semiconductor chips. During the experiment, we apply a laser with a fixed power and allow the sample to reach a steady-state thermal condition before turning off the laser and initiating a 5 s cooling phase. The time step for data acquisition is set to 0.125 s, resulting in 40 frames of thermal field data per experiment. To construct a comprehensive dataset for deriving thermal diffusivity parameters, we systematically adjust the laser’s position on the test sample, capturing a series of thermal field evolution data corresponding to different heat source locations. Furthermore, we refine the acquired thermal field evolution data by segmenting the cooling process into two distinct phases. Initially, the sample is allowed to cool for a duration $T_1$, after which only a short segment of the evolved final state thermal field, denoted as $T_2$, is selected as the dataset for time-inclusive thermal field retrodiction. For the estimation of thermal diffusivity in the actual inference model, we utilize the entire cooling period $T_1 + T_2$ for diffusivity derivation. In the subsequent thermal field retrodiction stage, however, only the data from the $T_2$ time period is used to reconstruct the thermal field evolution in a backward manner. From this, we denote the dataset consisting of the thermal field evolution over the entire cooling period as dataset1, and the evolution data including only the final state thermal field as dataset2. The retrodictive thermal field is then compared against the actual thermal field evolution to evaluate the inference accuracy of our model.

## Supplementary Material

nwag287_Supplemental_File
