# Supplementary material for: Learning to reverse thermal diffusion
Source: Natl Sci Rev. 2026 May 15;13(13):nwag287. doi: 10.1093/nsr/nwag287 (PMC13458607; doi:10.1093/nsr/nwag287)
Supplement: Supplementary file 1 [file nwag287_supplemental_file.docx]

**Supplementary Note 1: Analysis of the Temporal Derivative Estimator (Net2) in TE-Net**

In the main text, the Thermal Field Evolution Network (TE-Net) utilizes two distinct sub-networks: Net1 for deriving the spatial distribution of material parameters, and Net2 for estimating the temporal dynamics. Specifically, Net2 was designed to provide the time derivative of the temperature field to assist Net1 in estimating thermal diffusivity. We clarify here the theoretical motivation for Net2 and its practical role in our current experimental scope.

The standard finite-difference time marching schemes, as explicitly formulated in Eqs. (S2) and (S3), approximate the temporal derivative linearly over a discrete time step $\Delta t$.While mathematically rigorous, the precision of this explicit approximation is inherently bottlenecked by the sampling frequency (i.e., the frame rate of the infrared imaging equipment). At lower frame rates or in the presence of high-frequency measurement noise, the simple finite-difference approximation $\left( T^{\left( t+1 \right)}-T^{\left( t \right)} \right)/\Delta t$can introduce numerical instability and artifacts into the parameter inference process. Net2 was conceptualized as a continuous, learnable regularizer designed to extract a "true", noise-resilient temporal gradient directly from the spatiotemporal data manifold, thus decoupling the derivative accuracy from the hardware's sampling limitations.

Consequently, while Net2 offers a theoretically more robust and continuous gradient estimation—which remains highly valuable for potential future applications involving ultra-sparse data, extremely low frame rates, or highly transient heat sources—the explicit finite-difference scheme implicitly embedded in the loss function (Eq. (S4)) is practically sufficient for the sampling rates and materials used in this study. For the sake of model simplicity and computational efficiency in similar stable diffusion scenarios, the TE-Net framework can effectively converge and function without the strict necessity of the Net2 module.

**Supplementary Note 2: Derivation of Thermal Parameters (physics)**

For a non-homogeneous material, during its cooling process after being heated, in addition to the heat conduction within the material itself, the convective heat transfer with the surrounding air is also a significant influencing factor. Therefore, we can obtain the heat transfer governing Eq. (S1).

$$\begin{aligned} \rho c_{p}\frac{\partial T}{\partial t}-\nabla\cdot\left( \kappa\nabla T \right)=h\left( T_{e}-T \right)/d\#\left( S1 \right) \end{aligned}$$

$$\begin{aligned} T_{t}=\frac{\partial T}{\partial t}=\frac{h\left( T_{e}-T \right)/d+\nabla\cdot\left( \kappa\nabla T \right)}{\rho c_{p}}\#(S2) \end{aligned}$$

In the equation, $h$ represents the convective heat transfer coefficient, $d$ denotes the characteristic dimension (which, in this case, corresponds to the material thickness), $\rho$ is the density, $c_{p}$ is the specific heat capacity at constant pressure, $\kappa$ is the thermal conductivity, and $T_{e}$is the ambient temperature. Below, we briefly outline the steps for forward propagation using finite differences. By employing the explicit forward time, central space (FTCS) method, we derive the forward difference approximation for the time derivative as follows:

$$\begin{aligned} \frac{\partial T}{\partial t}=\frac{T_{x,y}^{t+1}-T_{x,y}^{t}}{\Delta t}\#\left( S3 \right) \end{aligned}$$

Substituting Eq. (2) into Eq. (3) yields:

$$\begin{aligned} T_{x,y}^{t+1}=\Delta\text{t}\left[ \frac{h\left( T_{e}-T \right)/d+\nabla\cdot\left( \kappa\nabla T \right)}{\rho c_{p}} \right]+T_{x,y}^{t}\#\left( S4 \right) \end{aligned}$$

This approach offers the advantage of eliminating the need for temporal calculations in the data, as computations are performed solely in the spatial domain, thereby reducing the complexity of the model. Eq. (S4) represents the time-stepping equation, which allows us to determine the temperature at time step $t+1$ given the temperature at time step $t$. We initiate these calculations by applying the initial conditions specified at $t=0$. Ideally, once we have the temperature field at $t=0$, the spatial derivatives of the thermal diffusivity and the Laplacian term can be approximated using finite differences. Consequently, in theory, knowing the temperature field at $t=0$ enables us to extrapolate the temperature field at any subsequent time.

From Eq. (S4), it is evident that to derive the evolving thermal field, one must first obtain the temperature field and the partial derivatives of the thermal diffusivity. For discrete data, we employ central difference approximations to estimate the first and second partial derivatives of both quantities. The formulas for these approximations are provided below (only the $x$-component of the temperature field $T$ is shown; the other components can be derived similarly):

$$\begin{aligned} \frac{\partial T}{\partial x}=T_{x}=\frac{T_{x+1,y}^{t}-T_{x-1,y}^{t}}{2\Delta x}\#\left( S5 \right) \end{aligned}$$

$$\begin{aligned} \frac{\partial^{2}T}{\partial x^{2}}=T_{xx}=\frac{T_{x+1,y}^{t}-2T_{x,y}^{t}+T_{x-1,y}^{t}}{\left( \Delta x \right)^{2}}\#\left( S6 \right) \end{aligned}$$

However, a new issue immediately arises: the central difference method cannot be applied at the boundaries. Moreover, in practical scenarios, the detected object is not strictly a two-dimensional model, and heat dissipation at the boundaries differs from that at interior points. Therefore, it is necessary to separately consider the evolution equations for boundary conditions.

**Supplementary Note 3: Boundary condition (physics)**

According to Fourier's law of heat conduction, in the absence of internal heat generation, the heat flux entering a pixel should satisfy the following relationship with its temperature change:

$$\begin{aligned} \sum_{i} q_{i}S_{i}=\rho c_{p}V\frac{\partial T}{\partial t}\#\left( S7 \right) \end{aligned}$$

where $q$ denotes the heat flux density, $S$ represents the cross-sectional area through which the heat flows, $\rho$ is the density of the region under study, $C_{p}$ is the specific heat capacity at constant pressure, $V$ is the volume of the region of interest, $T$ is the temperature, and $t$ is time. The sign convention for $q$ is defined as follows: heat flow entering the region of interest is considered positive, while heat flow exiting the region is considered negative.

In the actual measured sample, the $z$-direction must also be taken into account. Assuming an adiabatic boundary condition between the sample and the base, the contribution of heat flow in this direction can be expressed as:

$$\begin{aligned} \sum_{i} q_{i}S_{i}=q_{-x}S_{-x}+q_{+x}S_{+x}+q_{-y}S_{-y}+q_{+y}S_{+y}+q_{+z}S_{+z}\#\left( S8 \right) \end{aligned}$$

Here, the coordinate system is defined such that the positive $x$-axis points to the right, and the positive $y$-axis points downward. For all pixel points, the heat flux in the $z$-direction can be expressed using Newton's law of cooling as follows:

$$\begin{aligned} q_{+z}S_{+\text{z}}=h\left( T_{\mathrm{ext}}-T \right)\Delta x\Delta y\#\left( S9 \right) \end{aligned}$$

where the lengths in the x- and y-directions are denoted as $\Delta x$ and $\Delta y$, respectively, and $T_{ext}$ represents the ambient temperature.

For boundaries adjacent to other pixels, the interfacial heat flux can be expressed as:

$$\begin{aligned} q_{n}S=-\kappa\frac{\partial T}{\partial n}S\#\left( S10 \right) \end{aligned}$$

where $n$ can represent either $x$ or $y$. In practical scenarios, we can transform the above differential equation into a finite difference form. Taking the heat flux at the $-x$ boundary as an example, the equation can be expressed as:

$$\begin{aligned} q_{-x}S_{-x}=-\kappa\frac{T_{n}-T_{n-1}^{x}}{\Delta x}\Delta y\Delta z\#\left( S11 \right) \end{aligned}$$

where $T_{n}$ represents the temperature of the $n$-th pixel under investigation, and $T_{n-1}^{x}$ denotes the temperature of the pixel adjacent to the $n$-th pixel in the negative $x$-direction. Here, $\Delta z$ represents the thickness in the $z$-direction. For a boundary in contact with the outside world, the interfacial heat flux can be written as (assuming that the $-x$ direction is in contact with the outside interface):

$$\begin{aligned} q_{-x}S_{-x}=h\left( T_{\mathrm{ext}}-T \right)\Delta y\Delta z\#\left( S12 \right) \end{aligned}$$

The temperature evolution expression for the corresponding pixel can be obtained by taking the corresponding term in Eq. (S8), bringing it in with Eqs. (S9, S11, S12) depending on the specific heat transfer boundary at the location, and finally bringing it into Eq. (S7). If for the left boundary ($x=0$ non-corner), we have:

$$\frac{\partial T}{\partial t}=\frac{1}{\rho c_{p}V}\left[ q_{-x}S_{-x}+q_{+x}S_{+x}+q_{-y}S_{-y}+q_{+y}S_{+y}+q_{+z}S_{+z} \right]=\frac{1}{\rho c_{p}V}\left[ h\left( T_{\mathrm{ext}}-T \right)\Delta y\Delta z+\kappa\frac{T_{n+1}^{x}-T_{n}}{\Delta x}\Delta y\Delta z-\kappa\frac{T_{n}-T_{n-1}^{y}}{\Delta y}\Delta x\Delta z+\kappa\frac{T_{n+1}^{y}-T_{n}}{\Delta y}\Delta x\Delta z+h\left( T_{\mathrm{ext}}-T \right)\Delta x\Delta y \right]=\frac{1}{\rho c_{p}}\left[ \frac{h\left( T_{\mathrm{ext}}-T \right)}{\Delta x}+\kappa\frac{T_{n+1}^{x}-T_{n}}{\left( \Delta x \right)^{2}}+\kappa\frac{T_{n+1}^{y}+T_{n-1}^{y}-2T_{n}}{\left( \Delta y \right)^{2}}+\frac{h\left( T_{\mathrm{ext}}-T \right)}{\Delta z} \right]$$

$$\begin{aligned} \#\left( S13 \right) \end{aligned}$$

**Boundary Effect**

We have implemented this at the code level before and after adding the boundary-specific restrictions, and found that without adding the above boundary condition restrictions, there will be an error in the thermal diffusivity derivation in the boundary region. This error will be cumulative in the subsequent inversion of the thermal field, as it is a time-inclusive evolution of the thermal field, so it is necessary to consider the boundary separately.

We use the simulation data mentioned in the main text were fed into the model considering and not considering the boundary to obtain Fig. 1 from the figure can be seen, before the correction, not only its boundary there is a significant error, and affects the middle of the results through the boundary correction after the results can be very well derived.

**Supplementary Note 4: Thermal field evolution derivation (physics)**

Starting from the unparameterized partial differential equation for heat transfer, we use the partial differential (PDE) model

$$\begin{aligned} \frac{\partial}{\partial t}u\left( x,y,t \right)=\Delta u\left( x,y,t \right)\#\left( S14 \right) \end{aligned}$$

where $u\left( x,y,t \right)$ can be written in the form of an evolutionary equation as

$$\begin{aligned} u\left( x,y,t \right)=F\left( t \right)u\left( x,y,t_{0} \right)\#\left( S15 \right) \end{aligned}$$

In the equation, $F\left( t \right)=\exp\left[ \left( t-t_{0} \right)\Delta\right]$ represents the evolution operator, which is given by the operator exponential function (Eq. (S21)). It is worth noting that we can also consider more general (pseudo-)differential operators to replace the Laplacian operator, enabling the description of more complex dissipative processes.

When applying Neumann boundary conditions, we solve the associated eigenvalue problem. By choosing Neumann boundary conditions ($\frac{\partial u}{\partial x}=\frac{\partial u}{\partial y}=0$), where the derivatives at the boundaries of the image frame are set to zero, we obtain:

$$\begin{aligned} \left. \begin{aligned} \frac{\partial}{\partial x}u\left( 0,y,t \right)=0,\frac{\partial}{\partial x}u\left( W,y,t \right)=0 \\ \frac{\partial}{\partial x}u\left( x,0,t \right)=0,\frac{\partial}{\partial x}u\left( x,H,t \right)=0 \end{aligned} \right\}Boundary conditions\#\left( S16 \right) \end{aligned}$$

$$\begin{aligned} u\left( x,y,t_{0} \right)=f\left( x,y \right)\#\left( S17 \right) \end{aligned}$$

In the equation, $W$ represents the width of the image, and $H$ denotes its height. In many cases, other boundary conditions can also be chosen without loss of generality.

For the positive definite and Hermitian (negative) Laplacian operator, the solution to the eigenvalue problem can be expressed as:

$$\begin{aligned} \begin{matrix} -\Delta\varphi_{j}\left( x,y \right)=\lambda_{j}\varphi_{j}\left( x,y \right),\mathrm{for}\left( x,y \right)\in\Omega\\ \frac{\partial\varphi_{j}\left( x,y \right)}{\partial x}=\frac{\partial\varphi_{j}\left( x,y \right)}{\partial y}=0,\mathrm{for}\left( x,y \right)\in\Omega\end{matrix}\#\left( S18 \right) \end{aligned}$$

The corresponding inner product yields orthogonal eigenfunctions $\varphi_{j}(\cdot)$, indicating that the associated operator is diagonalizable. If $\Omega$ is a rectangular domain, and we consider the problem in a Cartesian coordinate system, the eigenbasis (obtained by solving the aforementioned eigenvalue problem under the specified boundary conditions) forms a (separable) cosine basis.

$$\begin{aligned} \varphi_{n,m}\left( x,y \right)\sim\cos\left( \frac{\pi nx}{W} \right)\cos\left( \frac{\pi ny}{H} \right)\#\left( S19 \right) \end{aligned}$$

$$\begin{aligned} \lambda_{n,m}=\pi^{2}\left( \frac{n^{2}}{W^{2}}+\frac{m^{2}}{H^{2}} \right)\#\left( S20 \right) \end{aligned}$$

Write the result of the evolution operator $F\left( t \right)=\exp\left[ \left( t-t_{0} \right)\Delta\right]$ explicitly,

$$\begin{aligned} u\left( x,y,t \right)=\sum_{n=0}^{\infty} \sum_{m=0}^{\infty} A_{nm}e^{-\pi^{2}\left( \frac{n^{2}}{W^{2}}+\frac{m^{2}}{H^{2}} \right)\left( t-t_{0} \right)}\cos\left( \frac{\pi nx}{W} \right)\cos\left( \frac{\pi ny}{H} \right)\#\left( S21 \right) \end{aligned}$$

where $A_{nm}$ is the coefficient of the projection of the initial state $u\left( x,y,t_{0} \right)$ onto the eigenfunction $\varphi_{n,m}( x , y)$.

Under the cosine basis, we can describe the thermal field evolution through the eigenvalue evolution, if we can map the kernel integration operator to the frequency domain for processing, thus we can give the kernel integration operator a meaning in the physical level of the frequency domain. Let $CT$ be the cosine transform of a function $CT:D\to\mathcal{R}^{\mathcal{dv}}$ and $CT^{-1}$ is its inverse. Let $k_{\phi}\left( x,y,a\left( x \right),a\left( y \right) \right)=k_{\phi}\left( x-y \right)$, applying the convolution theorem, we find that:

$$\begin{aligned} \left( \mathcal{K}\left( a;\phi\right)v_{k} \right)\left( x \right)=CT^{-1}\left( CT\left( k_{\phi} \right) \cdot CT\left( v_{k} \right) \right)\left( x \right),\forall x\in D\#\left( S22 \right) \end{aligned}$$

The reason for this modification of the kernel integral operator is that, according to our derivation of the heat evolution Eq. (S13) from the partial differential equation for heat conduction with the Newman boundary conditions, the eigenbasis of the evolution equation is the cosine basis. It is only necessary to perform a cosine transformation for the input thermal field, and we can represent the evolution process in terms of the eigenvalues, but since this derivation does not contain material parameters such as the thermal diffusivity, we need to introduce into the evolution process the mapping of the material parameters derived in the previous step after the cosine variation, which we consider to be non-varying with time, and so we can multiply the cosine variation directly by the above equation, and so we get:

$$\begin{aligned} CT\left( k_{\phi} \right)=\exp\left[ \lambda_{n,m}\left( t-t_{0} \right) \right]CT\left( D \right)\#(S23) \end{aligned}$$

Combining the above derivations, we finally update evolution equation:

$$\begin{aligned} v_{k+1}\left( x \right)=\sigma\left( Wv_{k}\left( x \right)+CT^{-1}\left( \exp\left[ \lambda_{n,m}\left( t-t_{0} \right) \right]\cdot CT\left( D \right) CT\left( v_{k} \right) \right)\left( x \right) \right),\forall x\in D\#\left( S24 \right) \end{aligned}$$

**Supplementary Note 5: Training details**

Our model is generally divided into two parts: a thermal diffusivity derivation part and a thermal field inversion part. Writing down we detail the specific training details of each of the two separately.

Regardless of the part, we make the global thermal field information available to be learned by the model by moving the location of the heat source point, either the thermal evolution information or the thermal diffusivity information.

**Thermal diffusivity derivation**

The thermal diffusivity is derived by the whole cooling thermal field evolution, and its specific method is described in more detail in the main text, so we will not repeat it here, and then we mainly introduce the details of the training of TRO.

**Training of TRO**

Training TRO mainly divides the dataset into two parts, assuming that the end-state thermal field (complete cooling) is $T_{n}$, and we invert the thermal field of the previous moments by the last $m$ thermal fields. Because in the real situation, we have and only know the last $m$ thermal field evolution, we design the training method as follows: derive the $m$ thermal fields that actually move forward $x$ frames by the last m thermal fields, where only the loss function generated by the overlapping part is calculated. The following Fig. 3 demonstrates the training of the first frame. The loop is then repeated to get the initial heat field we want.

**Supplementary Note 6: Error calculation**

Since we did not find relevant topics to find some unified error calculation method or evaluation index, we simply calculate the error as:

$$\begin{aligned} Error=\frac{T_{\mathrm{dedcue}}-T_{\mathrm{true}}}{T_{\mathrm{true}}}\#\left( S25 \right) \end{aligned}$$

In this paper, all the error-related calculations use the above equation.

**Supplementary Note 7: Architecture of the Reference Model**

The reference model (Ref. model) evaluated in our comparative experiments is based on the standard Fourier Neural Operator (FNO) introduced by Li et al. at ICLR 2021. The FNO is a resolution-invariant deep learning architecture designed to map infinite-dimensional function spaces. It achieves this by parameterizing the integral kernel directly in the Fourier space, effectively framing the integral operator as a convolution.

Specifically, within each Fourier layer, the hidden representation $v_{k}$ is updated using the following operation:

$$\begin{aligned} v_{k+1}\left( x \right)=\sigma\left( Wv_{k}\left( x \right)+\mathcal{F}^{-1}\left( R_{\phi}\cdot\left( \mathcal{F}v_{k} \right) \right)\left( x \right) \right)\#\left( S26 \right) \end{aligned}$$

where $\mathcal{F}$ and $\mathcal{F}^{-1}$ denote the Fourier transform and its inverse, $R_{\phi}$ is a learnable complex-valued weight tensor applied to the truncated lower Fourier modes, $W$ is a local linear transformation, and $\sigma$ is a non-linear activation function.

While the standard FNO serves as a powerful and highly efficient baseline for learning spatiotemporal dynamics, it operates as a purely data-driven model. In the context of our study, the Ref. model is trained directly on the thermal field evolution data without any physical constraints. Unlike our proposed Time-Reversal Operator (TRO), the standard FNO lacks the integration of physical prior knowledge, specifically the spatial distribution of thermal diffusivity inferred by the TE-Net. Furthermore, it does not include the physically meaningful residual correction term ($W$) specifically designed in the TRO to compensate for 3D-to-2D projection discrepancies and complex multi-physical coupling effects. Therefore, the comparison between the Ref. model and the TRO effectively isolates and demonstrates the critical necessity of embedding physical priors for accurate, high-fidelity thermal field retrodiction.

**Fig. S1:** Different results obtained with and without considering boundary conditions.

**Fig. S2:** Different initial state temperature fields at different heat source points.

**Fig. S3:** TE-Net training details. With $T_{n}$ as the end-state thermal field and the thermal field data of the last m frames, retrodicting to obtain $T_{n-1}$ to $T_{n-m-1}$. The overlapping part is the $n-1$ to $n-m$, and only the loss of the overlapping portion is calculated.

**Fig. S4:** Chip retrodiction thermal field results in the initial state at different heat source points (right up). The top row is the result of the reasoning, and the bottom row is the actual thermal field situation.

**Fig. S5:** **a**, Photograph of the experimental platform physically taken, consisting of a laser emitter, an optical path and an infrared microscope. **b**, Photographing the 3D-printed perforated structure under an optical microscope, you can see that it has a concentric circular structure inside.
